# Survival effect of different lymph node staging methods on ovarian cancer: An analysis of 10 878 patients

**DOI:** 10.1002/cam4.1680

**Published:** 2018-08-18

**Authors:** Jieyu Wang, Jun Li, Ruifang Chen, Xin Lu

**Affiliations:** ^1^ Department of Gynecology Obstetrics and Gynecology Hospital of Fudan University Shanghai China

**Keywords:** epithelial ovarian cancer, log odds of positive lymph nodes, lymph node ratio, lymphadenectomy, resected lymph nodes

## Abstract

**Background:**

To compare the survival impact of several lymph node staging methods and therapeutic role of lymphadenectomy in patients with epithelial ovarian cancer who had undergone lymphadenectomy.

**Methods:**

Data were retrospectively collected from the Surveillance, Epidemiology, and End Results program between 1988 and 2013.

**Results:**

An increasing number of resected lymph nodes (RLNs) was associated with a significant improvement in survival of FIGO stage II and III disease. However, for FIGO stage IV patients, better survival was not significantly associated with a more extensive lymphadenectomy. A higher lymph node ratio (LNR) and log odds of positive lymph nodes (LODDS) were associated with poorer survival regardless of stage. Nevertheless, four‐category classification of LODDS was more suitable for stage IV patients when three‐category classification was compatible with stage I‐III disease. Multivariate analysis demonstrated that LODDS and LNR were significant independent prognostic factors, but not RLN classification.

**Conclusion:**

Sixteen to thirty RLNs are recommended for stage I disease. For stages II and III patients, the more lymph node excision, the better the prognosis. However, lymphadenectomy was nonessential for stage IV patients. Considering staging methods, for stages II and III patients, three‐category classification of LODDS was recommended to evaluate the prognosis. For stage I and IV, three‐category classification of positive LNR was idoneous.

## 1 INTRODUCTION

Ovarian cancer is the fifth most common cancer and the fourth most common cause of cancer death. It is commonly diagnosed after menopause.^1^ Epithelial ovarian cancer (EOC) represents approximately 90% of ovarian cancers. The standard treatment of EOC includes primary cytoreduction surgery, accompanied by a platinum‐based chemotherapy.

Primary cytoreductive surgery is the primary treatment of EOC, and an optimal surgical outcome influences the prognosis of patients.^2^ Cytoreductive surgery can effectively excise the metastatic lesion, which is caused by transcoelomic spread in traditional view. However, recent reports^3^, ^4^, ^5^, ^6^ have indicated that dissemination through lymph nodes and vessels may be of equal importance to the traditional route. Despite the definite advantage of cytoreductive surgery, the benefit of lymphadenectomy in EOC remains controversial,^7^, ^8^ especially in different International Federation of Gynecology and Obstetrics (FIGO) stages.

Retrospective study has indicated that systematic lymphadenectomy can improve the 5‐year overall survival (OS) in advanced‐stage EOC, but not in the early stage or in patients with residual tumors ≤2 cm.^9^ Therefore, lymphadenectomy may only benefit the prognosis of ovarian cancer patients in advanced ovarian cancer patients with complete intraperitoneal debulking.^10^ Chan et al^11^ also demonstrated that in advanced EOC, the extent of lymphadenectomy affects the disease‐specific survival benefit. However, a prospective clinical trial demonstrated that a systematic lymph node dissection had no OS benefit when compared with a selected lymphadenectomy in advanced EOC.^12^ In early‐stage EOC, the data were also controversial. Contrary to Gao and Svolgaard,^9^, ^13^ who supported that lymphadenectomy does not yield survival benefits in early‐stage EOC, Chan^14^ found that lymphadenectomy was a significant prognostic factor for improved survival in stage I nonclear cell ovarian cancers.

Therefore, increasing attention has been paid to lymph node status, which can be evaluated by the number of resected lymph nodes (RLNs),^15^ lymph node ratio (LNR),^16^, ^17^, ^18^ and the log odds of positive lymph nodes (LODDS).^19^, ^20^, ^21^, ^22^, ^23^, ^24^ Compared to a single parameter, LNR exhibits its advantage in breast,^25^, ^26^, ^27^, ^28^ pancreatic,^19^ colon,^29^, ^30^, ^31^, ^32^ and ovarian^16^, ^18^ cancers. Another parameter, LODDS, showed superiority in predicting outcomes in breast,^20^ pancreatic,^19^, ^21^ colorectal,^22^, ^23^ and ovarian^24^ cancers.

Because the prior studies have been commonly limited by a small sample size, further studies are needed to explore the value of lymph node status in EOC patients with different stages. We performed a large population‐based database investigation of the prognostic value of RLNs, LNR, and LODDS in ovarian cancer patients with different stages, which may decrease the selection biases that are associated with small sample size. In this study, 10 878 EOC patients with RLN information were analyzed to determine the different potential role of LNs, LNR, and LODDS.

## 2 METHODS

Demographic, clinicopathologic, and survival information were generated from the Surveillance, Epidemiology, and End Results (SEER) database (http://seer.cancer.gov/). All pathologically confirmed and surgically treated EOC patients from 1 January 1988 to 31 December 2013 were identified. The following were the inclusion criteria: (a) Lymph nodes status were available, (b) pathology limited to epithelial ovarian cancer, (c) only one primary cancer in the patient's lifetime, (d) the primary site and morphology were ovarian, (e) survival time was available, and (f) grade was clear. A total of 10 878 patients were finally obtained. Patients were excluded if they had incomplete medical records, pathology, or outcome data.

Resected lymph nodes were used to evaluate lymphadenectomy extent, which were divided into six groups: one lymph node removed; two or three lymph nodes removed; four or five lymph nodes removed; six to 15 lymph nodes removed; 16‐30 lymph nodes removed; and 31 or more lymph nodes removed. Lymph node status was characterized by LNR and LODDS. LNR is the ratio of positive lymph nodes (PLNs) to RLNs. LODDS is defined as the log of odds between PLNs and the number of negative nodes. LNR and LODDS were first divided into a lot of groups. Through analysis, we found that there was little difference among some classifications. We amalgamated these classifications. Finally, LNR was subdivided into three groups to meet proportionality assumptions: 0 to <10%, 10% to <40%, and 40%‐100%. LODDS were grouped as follows: LODDS< −1.0, −1.0 ≤ LODDS<−0.5, −0.5 ≤ LODDS<0, LODDS≥0.

Survival was estimated by the Kaplan‐Meier method and assessed by the log‐rank test. OS was defined as the time between diagnosis and death due to any cause or censored at the last follow‐up time if no events had occurred. Cancer‐specific survival (CSS) was defined as the time between diagnosis and cancer‐specific death. To investigate the significance of RLNs, LNR, and LODDS, multivariate analysis was performed using the Cox proportional hazards model after adjusting for other patient features, including age, race, tumor size, and grade in different FIGO stages. Analyses were performed using the SPSS statistical software package, version 19.0 (IBM Corporation, Armonk, NY, USA) and the R version 3.3.0 software (Institute for Statistics and Mathematics, Vienna, Austria; http://www.r-project.org). All tests were two‐tailed, and statistical significance remained conventionally defined as *P *<* *0.05 in all other cases.

## 3 RESULTS

**Figure 1:**
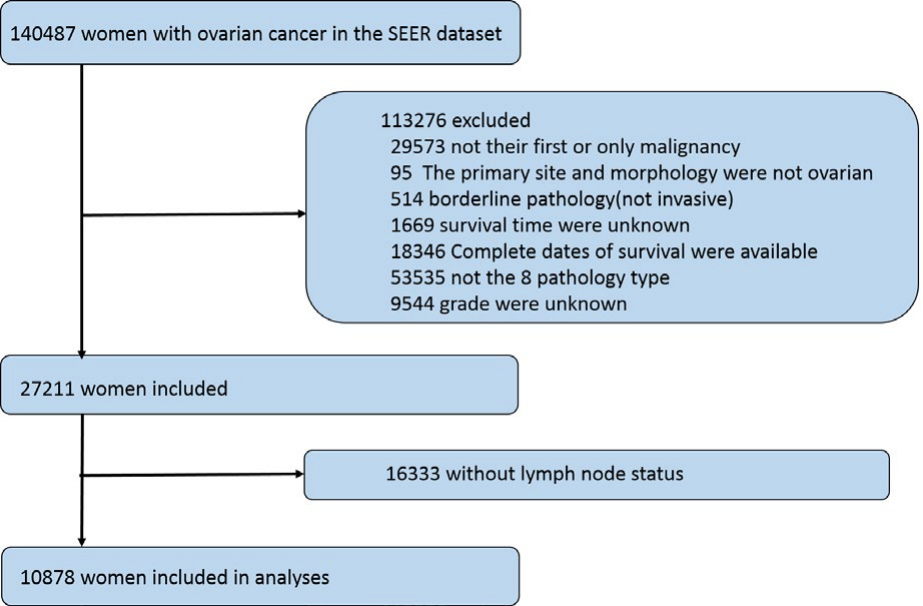
Construction of study population

The SEER dataset included 140 487 women who were diagnosed with ovarian cancer. A total of 113 276 women were excluded, 29 573 cases because ovarian cancer was not their first or only malignancy, 95 cases because the primary site was not ovarian, and/or morphology was not epithelial ovarian cancer, 514 cases due to borderline pathology, 20 015 patients with incomplete survival information, 53 535 cases because of pathology type, and 9544 women without grade information. As this study focused on analyzing lymph node status, 16 333 patients without information about lymph node harvest were also excluded. Therefore, the final analysis comprised 10 878 cases (Figure 1).

Table 1 summarized the demographic and clinicopathologic characteristics according to the number of RLNs, LNR, and LODDS classification. The median patient age was 57.0 years (range: 15‐96), 84.8% were Caucasian, and 51.8% had serous histology. The majority of patients were stage I (34.0%) or stage III (37.4%) at the time of surgery. A large proportion of patients had grade 3 disease (59.9%), with 14.4% and 25.7% of patients having grades 1 and 2 disease, respectively. The median survival time was 44 months (range: 0‐311). The median number of RLNs in the entire cohort was 10 (range: 1‐90). The number of patients with 1, 2‐3, 4‐5, 6‐15, 16‐30, and more than 31 RLNs was 1104 (10.1%), 1366 (12.6%), 1016 (9.8%), 3755 (34.5%), 2624 (24.1%), and 968 (8.9%), respectively. There were 5575 patients in the LODDS<−1 group, 2014 cases in −1 ≤ LODDS<−0.5, 1386 cases in −0.5 ≤ LODDS<0, and 1890 cases in LODDS≥0. Among all the patients, the majority of LNRs were in the 0‐<10% (70.3%) group, with 11.3% in the 10% to <40% group, and 18.4% in 40%‐100% group.

**Table 1:** Clinical and pathological characteristics of patients stratified by (a) the number of RLNs; (b) LNR and LODDS

(a)							
	Total number of resected lymph nodes						Total
	1 node	2‐3 nodes No. (%)^a^	4‐5 nodes No. (%)^a^	6‐15 nodes No. (%)^a^	16‐30 nodes No. (%)^a^	≥31 nodes No. (%)^a^	
Age							
Mean	59.7 ± 0.4	58.8 ± 0.4	57.8 ± 0.4	57.0 ± 0.2	56.3 ± 0.2	54.9 ± 0.4	57.2 ± 0.1
Median (range)	59.0 (16‐91)	59.0 (18‐90)	57.0 (18‐96)	56.0 (15‐92)	56.0 (16‐90)	54.5 (21‐88)	57.0 (15‐96)
Tumor size							
≤1 cm	27 (0.3%)	23 (0.3%)	14 (0.2%)	77 (1.0%)	68 (0.9%)	15 (0.2%)	224 (2.8%)
1‐5 cm	152 (1.9%)	201 (2.5%)	143 (1.8%)	536 (6.7%)	383 (4.8%)	161 (2.0%)	1576 (19.8%)
5‐10 cm	213 (2.7%)	291 (3.6%)	257 (3.2%)	855 (10.7%)	640 (8.0%)	211 (2.6%)	2467 (30.9%)
10‐20 cm	271 (3.4%)	367 (4.6%)	304 (3.8%)	1101 (13.8%)	779 (9.8%)	282 (3.5%)	3104 (38.9%)
>20 cm	48 (0.6%)	62 (0.8%)	59 (0.7%)	228 (2.9%)	153 (1.9%)	54 (0.7%)	604 (70.6%)
Race							
Caucasians	949 (8.7%)	1129 (10.4%)	880 (8.1%)	3218 (29.6%)	2230 (20.5%)	819 (7.5%)	9225 (84.8%)
Black	75 (0.7%)	105 (1.0%)	67 (0.6%)	171 (1.6%)	104 (1.0%)	29 (0.3%)	551 (5.1%)
African	13 (0.1%)	10 (0.1%)	9 (0.1%)	16 (0.1%)	6 (0.1%)	6 (0.1%)	60 (0.6%)
Asians^a^	63 (0.6%)	114 (1.0%)	95 (0.9%)	325 (3.0%)	256 (2.4%)	104 (1.0%)	957 (8.8%)
Pacific islander	4 (0.0%)	6 (0.1%)	4 (0.0%)	16 (0.1%)	15 (0.1%)	6 (0.1%)	51 (0.5%)
Others^b^	0 (0.0%)	0 (0.0%)	4 (0.0%)	5 (0.0%)	7 (0.1%)	2 (0.0%)	18 (0.2%)
Unknown	0 (0.0%)	2 (0.0%)	2 (0.0%)	4 (0.0%)	6 (0.1%)	2 (0.0%)	16 (0.1%)
Stage of disease							
Stage I	185 (1.7%)	347 (3.2%)	328 (3.0%)	1439 (13.2%)	1061 (9.8%)	343 (3.2%)	3703 (34.0%)
Stage II	102 (0.9%)	184 (1.7%)	160 (1.5%)	521 (4.8%)	360 (3.3%)	117 (1.1%)	1444 (13.3%)
Stage III	530 (4.9%)	546 (5.0%)	408 (3.8%)	1309 (12.0%)	893 (8.2%)	383 (3.5%)	4069 (37.4%)
Stage IV	287 (2.6%)	289 (2.7%)	165 (1.5%)	486 (4.5%)	310 (2.8%)	125 (1.1%)	1662 (15.3%)
Grade of disease							
Grade 1	95 (0.9%)	166 (1.5%)	157 (1.4%)	595 (5.5%)	410 (3.8%)	145 (1.3%)	1568 (14.4%)
Grade 2	259 (2.4%)	332 (3.1%)	260 (2.4%)	1019 (9.4%)	696 (6.4%)	225 (2.1%)	2791 (25.7%)
Grade 3	750 (6.9%)	868 (8.0%)	644 (5.9%)	2141 (19.7%)	1518 (14.0%)	598 (5.5%)	6519 (59.9%)
Histology							
Serous	732 (6.7%)	807 (7.4%)	565 (5.2%)	1828 (16.8%)	1232 (11.3%)	475 (4.4%)	5639 (51.8%)
Clear cell	75 (0.7%)	115 (1.1%)	114 (1.0%)	456 (4.2%)	365 (3.3%)	125 (1.1%)	1249 (11.5%)
Mucinous	69 (0.6%)	101 (0.9%)	78 (0.7%)	342 (3.1%)	180 (1.7%)	65 (0.6%)	835 (7.7%)
Endometrioid	205 (1.9%)	323 (3.0%)	275 (2.5%)	1051 (9.7%)	806 (7.4%)	282 (2.6%)	2942 (27.17%)
Carcinosarcoma	12 (0.1%)	8 (0.1%)	13 (0.1%)	35 (0.3%)	28 (0.3%)	13 (0.1%)	109 (1.0%)
Undifferentiated	11 (0.1%)	12 (0.1%)	16 (0.1%)	43 (0.4%)	14 (0.1%)	8 (0.1%)	104 (1.0%)

(b)								
	LNR			LODDS				Total
	0%‐10%	10%‐40%	40%‐100%	LODDS<−1	−1 ≤ LODDS<−0.5	−0.5 ≤ LODDS<0	LODDS≥0	
Age								
Mean	56.5 ± 0.1	57.9 ± 0.4	59.7 ± 0.3	55.8 ± 0.2	57.8 ± 0.3	58.5 ± 0.3	59.8 ± 0.4	57.2 ± 0.1
Median (range)	56.0 (15‐96)	58.0 (17‐89)	60.0 (17‐91)	55.0 (15‐96)	58.0 (18‐90)	58.0 (16‐91)	59.0 (17‐91)	57.0 (15‐96)
Tumor size								
≤1 cm	177 (2.2%)	19 (0.2%)	28 (0.4%)	135 (1.7%)	34 (0.4%)	29 (0.4%)	26 (0.3%)	224 (2.8%)
1‐5 cm	1077 (13.5%)	186 (2.3%)	312 (3.9%)	793 (9.9%)	280 (3.5%)	210 (2.6%)	292 (3.7%)	1575 (19.8%)
5‐10 cm	1691 (21.2%)	293 (3.7%)	481 (6.0%)	1261 (15.8%)	455 (5.7%)	293 (3.7%)	456 (5.7%)	2465 (30.9%)
10‐20 cm	2391 (30.0%)	289 (3.6%)	422 (5.3%)	1794 (22.5%)	556 (7.0%)	349 (4.4%)	403 (5.0%)	3102 (38.9%)
>20 cm	508 (6.4%)	48 (0.6%)	48 (0.6%)	389 (4.9%)	102 (1.3%)	69 (0.9%)	44 (0.5%)	604 (7.6%)
Race								
Caucasians	6440 (59.3%)	1056 (9.7%)	1718 (15.8%)	4715 (43.4%)	1691 (15.6%)	1184 (10.9%)	1624 (14.9%)	9214 (84.8%)
Black	349 (3.2%)	81 (0.7%)	120 (1.1%)	208 (1.9%)	131 (1.2%)	96 (0.9%)	115 (1.1%)	550 (5.1%)
African	35 (0.3%)	12 (0.1%)	13 (0.1%)	22 (0.2%)	12 (0.1%)	14 (0.1%)	12 (0.1%)	60 (0.6%)
Asians	742 (6.8%)	77 (0.7%)	137 (1.3%)	571 (5.3%)	168 (1.5%)	88 (0.8%)	129 (1.2%)	956 (8.8%)
Pacific islander	41 (0.4%)	3 (0.0%)	7 (0.1%)	35 (0.3%)	5 (0.0%)	4 (0.0%)	7 (0.0%)	51 (0.5%)
Others	16 (0.1%)	0 (0.0%)	2 (0.0%)	12 (0.1%)	4 (0.0%)	0 (0.0%)	2 (0.0%)	18 (0.2%)
Unknown	15 (0.1%)	0 (0.0%)	1 (0.0%)	12 (0.1%)	3 (0.0%)	0 (0.0%)	1 (0.0%)	16 (0.1%)
FIGO stage of disease								
Stage I	3666 (33.7%)	29 (0.3%)	8 (0.1%)	2975 (27.4%)	525 (4.8%)	195 (1.8%)	8 (0.1%)	3703 (34.1%)
Stage II	1344 (12.4%)	37 (0.3%)	59 (0.5%)	1015 (9.3%)	267 (2.5%)	101 (0.9%)	57 (0.6%)	1440 (13.3%)
Stage III	1997 (18.4%)	859 (7.9%)	1208 (11.1%)	1263 (11.6%)	906 (8.3%)	761 (7.0%)	1134 (9.5%)	4064 (37.4%)
Stage IV	631 (5.8%)	304 (2.8%)	723 (6.7%)	322 (3.0%)	316 (2.9%)	329 (3.0%)	691 (6.4%)	1658 (15.3%)
Grade of disease								
Grade 1	1424 (13.1%)	76 (0.7%)	67 (0.6%)	114 (10.3%)	272 (2.5%)	118 (1.1%)	63 (0.5%)	1567 (14.4%)
Grade 2	2241 (20.6%)	205 (1.9%)	342 (3.1%)	1664 (15.3%)	497 (4.6%)	313 (2.9%)	314 (2.9%)	2788 (25.7%)
Grade 3	3973 (36.6%)	948 (8.7%)	1589 (14.6%)	2797 (25.7%)	1245 (11.5%)	955 (8.8%)	1513 (5.2%)	6510 (59.9%)
Histology								
Serous	3054 (28.1%)	930 (8.6%)	1644 (15.1%)	1987 (18.3%)	1127 (10.4%)	966 (8.8%)	1548 (14.3%)	5628 (51.8%)
Clear cell	1066 (9.8%)	90 (0.8%)	93 (0.9%)	869 (8.0%)	192 (1.8%)	97 (0.9%)	91 (0.8%)	1249 (11.5%)
Mucinous	760 (7.0%)	38 (0.3%)	37 (0.3%)	576 (5.3%)	154 (1.4%)	71 (0.7%)	34 (0.3%)	835 (7.7%)
Endometrioid	2626 (24.1%)	136 (1.2%)	178 (1.6%)	2056 (18.9%)	490 (4.6%)	221 (2.0%)	173 (1.5%)	2940 (27.1)
Carcinosarcoma	75 (0.7%)	15 (0.1%)	19 (0.2%)	50 (0.5%)	27 (0.2%)	14 (0.1%)	18 (0.2%)	109 (1.0%)
Undifferentiated	57 (0.5%)	20 (0.2%)	27 (0.2%)	37 (0.3%)	24 (0.2%)	17 (0.2%)	26 (0.3%)	104 (1.0)

a Asians were defined as Chinese, Japanese, Korean, Vietnamese, and Filipino.

b Others were defined as all other race/ethnicity parameters.

**Figure 2:**
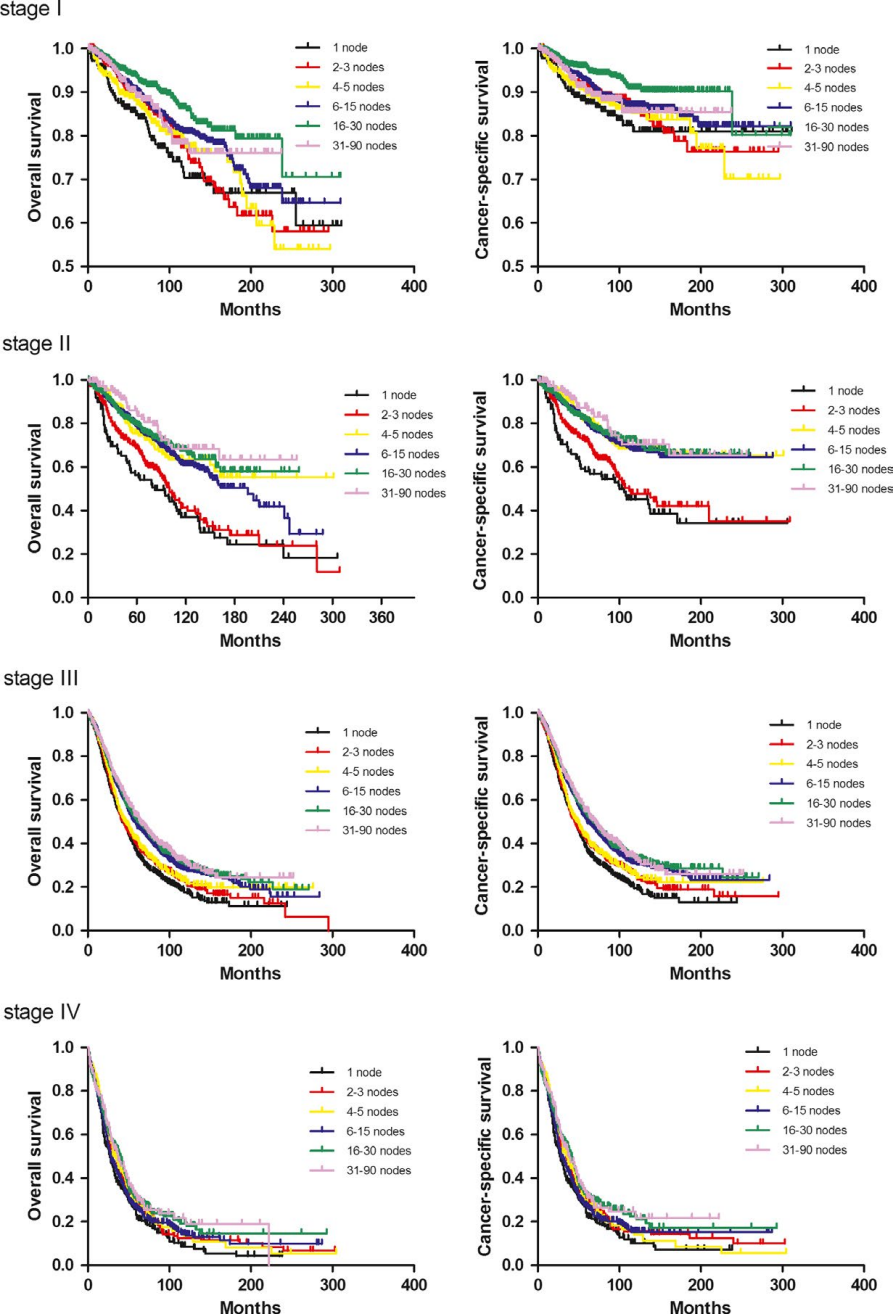
Kaplan‐Meier analysis of overall survival of RLNs according to FIGO stages

To evaluate the effect of the extent of lymph node dissection, our study cohort was divided into six groups: Patients who had 1, 2‐3, 4‐5, 6‐15, 16‐30, and ≥31 nodes were reported. We determined whether the influence of RLNs on CSS and OS was modified by FIGO stages. When FIGO stages were studied separately, the beneficial effect of lymphadenectomy with different extent was not observed for all FIGO stages (Table 2a; Figure 2). The results indicated that in FIGO stages II and III, an increasing numbers of lymph nodes were associated with a significant improvement in 5‐y and 10‐y CSS or OS. The findings of 1, 2‐3, 4‐5, 6‐15, 16‐30, and ≥31 lymph nodes were associated with 10‐y OS of 37.1%, 41.3%, 63.5%, 60.9%, 67.7%, and 68.4% in FIGO II stage, and 17.6%, 22.4%, 23.0%, 28.0%, 29.9%, and 30.7% in FIGO III stage, respectively (*P* < 0.0001). However, for 3703 patients with stage I disease, the optimal survival rate was achieved in the 16‐30 RLNs group, instead of the ≥31 group (OS: *P *=* *0.0371; CSS: *P *=* *0.0154). In 1658 EOC cases with stage IV, better OS was not found to be significantly associated with a more extensive lymphadenectomy (*P *=* *0.0538).

**Table 2:** Cancer‐specific and overall survival according to (a) the number of RLNs in different FIGO stages; (b) LNR and LODDS in different FIGO stages

(a)							
	The number of RLNs						*P* value
	1 node	2‐3 nodes	4‐5 nodes	6‐15 nodes	16‐30 nodes	≥31 nodes	
FIGO stage I							
5‐year CSS	87.5%	91.1%	89.3%	92.4%	95.6%	89.9%	**0.0033**
10‐year CSS	81.0%	86.7%	85.3%	86.9%	90.8%	85.4%	
5‐year OS	84.8%	89.0%	87.3%	90.4%	93.5%	88.9%	**0.0003**
10‐year OS	70.4%	77.3%	77.4%	80.8%	86.4%	78.3%	
FIGO stage II							
5‐year CSS	58.1%	71.8%	81.3%	80.1%	81.3%	87.4%	**<0.0001**
10‐year CSS	45.2%	47.7%	68.5%	67.6%	72.2%	70.4%	
5‐year OS	56.4%	68.5%	75.4%	78.6%	79.3%	84.8%	**<0.0001**
10‐year OS	37.1%	41.3%	63.5%	60.9%	67.7%	68.4%	
FIGO stage III							
5‐year CSS	36.9%	41.7%	43.1%	50.2%	52.9%	54.9%	**<0.0001**
10‐year CSS	19.5%	25.6%	25.9%	31.1%	32.9%	32.6%	
5‐year OS	35.5%	39.7%	41.9%	47.9%	50.4%	52.5%	**<0.0001**
10‐year OS	17.6%	22.4%	23.0%	28.0%	29.9%	30.7%	
FIGO stage IV							
5‐year CSS	22.4%	30.7%	28.1%	27.2%	32.3%	32.3%	**0.0390**
10‐year CSS	10.1%	15.5%	14.1%	15.2%	22.5%	24.7%	
5‐year OS	21.4%	29.3%	27.1%	26.2%	30.5%	31.1%	0.0538
10‐year OS	8.6%	12.4%	13.6%	14.3%	19.2%	21.6%	

(b)									
	LNR (%)			*P* value	LODDS (%)				*P* value
	0‐10	10‐40	40‐100		LODDS<−1	−1 ≤ LODDS<−0.5	−0.5 ≤ LODDS<0	LODDS≥0	
FIGO stage I									
5‐year CSS	92.7	72.1	46.9	**<0.0001**	93.4	89.9	87.1	46.9	**<0.0001**
10‐year CSS	87.6	68.1	46.9		88.4	84.9	81.2	46.9	
5‐year OS	90.8	62.1	37.5	**<0.0001**	91.7	86.9	84.0	45.0	**<0.0001**
10‐year OS	81.0	58.6	25.0		82.8	75.8	70.8	30.0	
FIGO stage II									
5‐year CSS	80.5	70.3	38.6	**<0.0001**	82.7	77.1	65.9	36.3	**<0.0001**
10‐year CSS	67.3	59.0	19.9		71.2	58.1	52.7	19.1	
5‐year OS	78.0	70.3	38.7	**<0.0001**	80.7	72.6	64.9	36.7	**<0.0001**
10‐year OS	61.2	53.1	18.5		66.1	49.9	43.9	19.2	
FIGO stage III									
5‐year CSS	54.3	49.0	35.0	**<0.0001**	58.4	52.0	43.1	34.5	**<0.0001**
10‐year CSS	34.0	29.1	19.1		37.5	30.9	26.3	18.1	
5‐year OS	52.5	46.6	32.8	**<0.0001**	56.5	49.9	41.1	32.5	**<0.0001**
10‐year OS	31.4	26.3	16.4		34.7	28.0	24.2	15.4	
FIGO stage IV									
5‐year CSS	39.5	29.4	18.1	**<0.0001**	41.9	38.5	26.1	18.6	**<0.0001**
10‐year CSS	24.0	15.0	9.4		26.1	23.1	12.6	9.5	
5‐year OS	37.8	28.6	17.0	**<0.0001**	39.8	37.2	25.5	17.6	**<0.0001**
10‐year OS	20.2	13.9	8.2		26.1	23.1	12.6	9.9	

Significant values are in bold.

**Figure 3:**
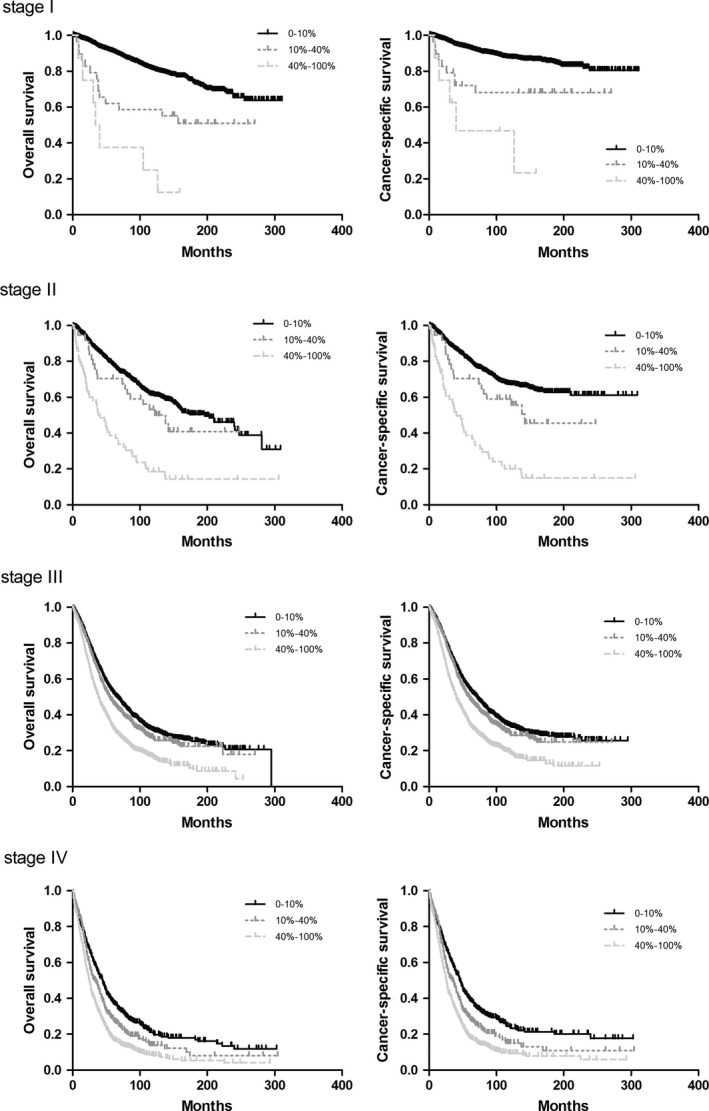
Kaplan‐Meier analysis of overall survival of LNR according to FIGO stages

**Figure 4:**
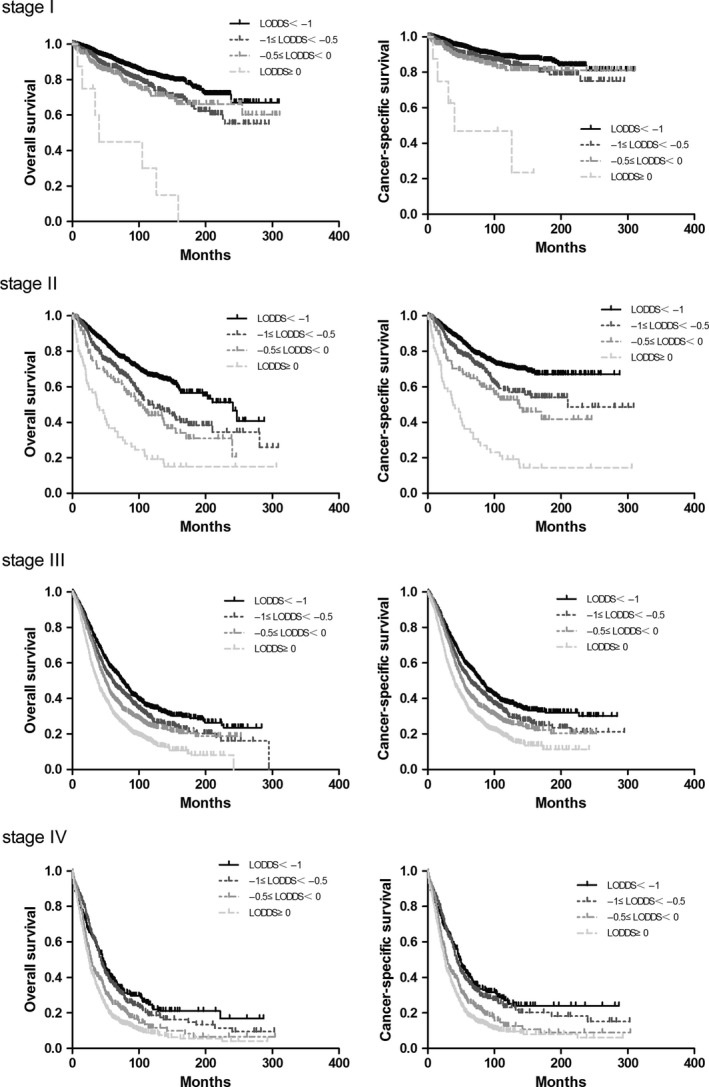
Kaplan‐Meier analysis of overall survival of LODDS according to FIGO stages

The effects of LNR and LODDS are shown in Table 2b and Figure 3. The 5‐ and 10‐y CSS and OS rates were found to be significantly decreased when more positive lymph nodes appeared within all FIGO stages. In an analysis of 5504 patients who had FIGO stages II and III disease, the 10‐y OS was 61.2% for patients with an LNR of 0 to <10%, 53.1% for those with an LNR of 10% to <40%, and 18.5% for those with an LNR of 40%‐100% (*P *<* *0.0001). In FIGO II, the 10‐y OS rates were 31.4%, 26.3%, and 16.4% for groups in which LNR was between 0 and 10%, between 10 and 40%, and more than 40%, respectively (*P* <0.0001). Notably, the 10‐y OS rates in FIGO I were 81.0%, 68.1%, and 46.9% of three‐category LNR (*P* <0.0001), which indicated positive lymph nodes, especially the 0%‐10% and 10%‐40% groups, were more important in FIGO I. Additionally, the effect of LODDS was investigated in subgroups of patients with FIGO stages I‐IV disease (Figure 4). Generally, a higher LODDS was significantly associated with poorer CSS (*P *<* *0.0001) and OS (*P *<* *0.0001). Nonetheless, differences exist in various FIGO stages. The four‐category system is suitable for FIGO IV patients, with 10‐y OS of 26.1%, 23.1%, 12.6%, and 9.9%, respectively. Three categories (LODDS<−1, −1 ≤ LODDS<0, and LODDS≥0) are compatible with FIGO stages I‐III.

On multivariate analysis, LNR persisted as a significant and independent prognostic factor of OS (Table 3a) and CSS (Table 3b), regardless of the stage of the disease. The hazard ratios (HRs) of LNR on OS in FIGO stages I‐IV were 2.569, 1.670, 1.234, and 1.259, respectively. LODDS was also a significant prognostic factor, which was associated with a poorer CSS and OS.

**Table 3:** Multivariate analysis of prognostic factors in (a) overall survival; (b) cancer‐specific survival

Prognostic factor	FIGO I			FIGO II			FIGO III			FIGO IV		
	Hazard ratio	95% confidence interval	*P*	Hazard ratio	95% confidence interval	*P*	Hazard ratio	95% confidence interval	*P*	Hazard ratio	95% confidence interval	*P*
(a)												
Race	1.106	1.003‐1.221	**0.044**	0.914	0.808‐1.034	0.152	0.999	0.936‐1.065	0.969	0.965	0.879‐1.060	0.458
Age	1.274	1.223‐1.328	**0.000**	1.139	1.089‐1.191	**0.000**	1.097	1.074‐1.120	**0.000**	1.069	1.036‐1.104	**0.000**
Grade	1.296	1.126‐1.492	**0.000**	1.376	1.160‐1.632	**0.000**	1.338	1.215‐1.474	**0.000**	1.185	1.012‐1.389	**0.035**
Tumor size	1.047	1.020‐1.075	**0.001**	1.011	0.982‐1.041	0.464	0.988	0.975‐1.001	0.062	1.005	0.986‐1.025	0.608
RLNs	0.895	0.827‐0.969	**0.006**	0.818	0.757‐0.884	**0.000**	0.949	0.917‐0.982	**0.002**	0.966	0.920‐1.014	0.165
LNR	2.569	1.766‐3.737	**0.000**	1.671	1.385‐2.017	**0.000**	1.234	1.163‐1.310	**0.000**	1.295	1.191‐1.408	**0.000**
LODDS	1.423	1.229‐1.647	**0.000**	1.499	1.358‐1.654	**0.000**	1.184	1.141‐1.230	**0.000**	1.174	1.114‐1.238	**0.000**
(b)												
Race	1.074	0.952‐1.211	0.244	0.883	0.767‐1.018	0.086	0.995	0.931‐1.063	0.878	0.955	0.867‐1.052	0.353
Age	1.122	1.066‐1.181	**0.000**	1.074	1.021‐1.129	**0.005**	1.082	1.059‐1.105	**0.000**	1.065	1.032‐1.099	**0.000**
Grade	1.664	1.387‐1.996	**0.000**	1.453	1.195‐1.768	**0.000**	1.377	1.245‐1.524	**0.000**	1.188	1.009‐1.399	**0.039**
Tumor size	1.040	1.007‐1.074	**0.017**	1.009	0.976‐1.043	0.591	0.985	0.972‐0.999	**0.032**	1.002	0.982‐1.022	0.860
RLNs	0.899	0.814‐0.993	**0.036**	0.833	0.763‐0.910	**0.000**	0.947	0.915‐0.981	**0.003**	0.968	0.921‐1.017	0.200
LNR	2.589	1.632‐4.080	**0.000**	1.836	1.504‐2.242	**0.000**	1.221	1.148‐1.298	**0.000**	1.337	1.227‐1.456	**0.000**
LODDS	1.418	1.178‐1.706	**0.000**	1.561	1.402‐1.739	**0.000**	1.182	1.137‐1.229	**0.000**	1.186	1.124‐1.252	**0.000**

The bold values present p < 0.05.

In contrast, the number of lymph nodes resected was a significant prognostic factor only for FIGO I‐III on OS (HR: 0.895, 95% confidence interval [CI]: 0.827‐0.969 in stage I; HR: 0.818, 95% CI: 0.757‐0.884 in stage II; HR: 0.949, 95% CI: 0.917‐0.982 in stage III; *P *<* *0.001) and CSS (HR: 0.899, 95% CI: 0.814‐0.993 in stage I; HR: 0.833, 95% CI: 0.763‐0.910 in stage II; HR: 0.947, 95% CI: 0.915‐0.981 in stage III; *P *<* *0.001). In FIGO IV patients, RLNs had no significant effect on CSS (HR: 0.968, 95% CI: 0.921‐1.017, *P *=* *0.200) and OS (HR: 0.966, 95% CI: 0.920‐1.014, *P *=* *0.165), suggesting that not all stages of patients could benefit from lymphadenectomy. In addition, multivariate analysis also indicated that age and grade were significant prognostic factors for OS (Table 3) and CSS (Table 4).

## 4 DISCUSSION

Ovarian cancer is the most common cancer and has the highest death rate in female malignancy. Although surgery is definitely necessary in the treatment of EOC, the role of lymphadenectomy is controversial. In the present study, we investigated the prognostic value of RLNs, LNR, and LODDS in EOC patients with different disease stages. Our results showed that in different stages, the prognostic value of lymph node parameters was inconformity. In FIGO stage II and III, an increasing number of RLNs was associated with a significant improvement in survival. However, for stage I disease, the optimal survival rate was achieved in the 16‐30 RLNS group, and in stage IV patients, a more extensive lymphadenectomy did not yield survival benefits. In all disease stages, a higher LNR and LODDS were significantly associated with poorer survival. Furthermore, the two‐category system of LNR was suitable for FIGO stage II and III patients, and the three‐category system was suitable for FIGO I and IV patients. Three LODDS categories (LODDS<−1, −1 ≤ LODDS<0, and LODDS≥0) were compatible with FIGO I‐III disease. However, for FIGO IV patients, the four‐category system (LODDS<−1, −1 ≤ LODDS<−0.5, −0.5 ≤ LODDS<0, and LODDS≥0) is more suitable. Multivariate analysis demonstrated that LODDS and LNR were significant independent prognostic factors regardless of the disease stage, but not RLN classification.

Compared to the previous FIGO staging system, the revised FIGO staging system utilizes PLNs to characterize stage III.^33^, ^34^ However, because PLNs is not reflex the information of negative lymph nodes, it is not appropriate to make predictions. LNR, combining positive lymph nodes and resected number, should be utilized to reduce the potential bias.^18^ In stage IIIC ovarian cancer patients with lymphadenectomy, increasing LNR leads to significantly decreased OS,^18^, ^35^ which was similar to the results of our study. In our study, we demonstrated that, in FIGO stage III patients, LNR within 0 to ≤40% group had survival advantage over LNR more than 40% group. Furthermore, this two‐category system is also suitable for stage II disease. However, due to the significant difference of survival between LNR>0 to ≤10% and LNR>10 to ≤40%, dividing LNR into three parts (0 to ≤10%, 10% to ≤40%, and >40%) was more suitable in FIGO stage IV. Because the positive nodes were more common in advanced‐stage disease, studies about LNRs have always been focused on those patients. Therefore, the role of LNR in early‐stage disease was ambiguous. Our research demonstrated that classifying LNR into three parts (0 to ≤10%, 10% to ≤40%, and >40%) was associated with decreased OS in FIGO stage I. Based on these findings, our study recommends the adaptation of the two‐category system of LNR in FIGO stage II and III, and the three‐category system in FIGO stage I and IV. Through this method, the survival status of patients can be predicted more accurately.

Log odds of positive lymph nodes, as an emerging indicator of lymph node status, has been studied in various cancers.^19^, ^20^, ^21^, ^22^, ^23^ Little is known about the role of LODDS in ovarian cancer. Xu et al^24^found LODDS as an independent prognostic factor for predicting survival in patients with EOC regardless of the tumor stage. Although LODDS exhibits discriminatory ability in predicting survival, there is no study concerning the diverse role of LODDS in different FIGO stages. Our study demonstrated that in FIGO stage I to III patients, the three classification method (LODDS<−1, −1 ≤ LODDS<0, and LODDS≥0) could stratified patients clearly. However, in FIGO IV, classification of LODDS into four categories is more suitable for prediction.

The therapeutic value of lymphadenectomy in ovarian cancer remains controversial. In early stages, survival of patients with lymph node dissection was significantly better than that of patients without lymph node dissection,^36^ whereas, Maggioni et al^37^ reported that, in FIGO stage I‐II patients, there was no difference between women undergoing a node sampling and those who underwent a lymphadenectomy. In our study, we found that lymphadenectomy had diverse effects on stage I and II disease. In FIGO stage I, a large number of resected lymph nodes did not represent a better outcome. The patients with 16‐30 RLNs have the best prognosis. When RLNs numbered more than 30, the survival rate decreased. In FIGO stage II, the minimum number of RLNS for a good prognosis is 4, preferably up to 16. According to our study, in early‐stage ovarian cancer, the goal of the extent of lymphadenectomy is to remove 16‐30 lymph nodes. In advanced‐stage lymphadenectomy, there were also several opinions. Pereira^38^ reported that removing more than 40 lymph nodes may yield survival benefit. Iwase,^39^ however, indicated that systematic lymphadenectomy did not confer therapeutic benefits, which is similar to our results in FIGO stage IV. In FIGO III, we suggest that the number of RLNs should be more than 6.

In conclusion, our data suggest that the prognostic information provided by LNRs or LODDS should be deciphered according to patients’ FIGO stage. Flexible application of LNR or LODDS in different FIGO stages to characterize ovarian cancer patients might predict outcomes more precisely. Furthermore, taking FIGO stage into consideration when carrying out lymphadenectomy may avoid a lack of benefit in patients with ovarian cancer, which can further contribute to the individualized clinical decision. To prevent the bias inherent to the retrospective methodology, the results of this study need to be confirmed in future prospective studies.

## CONFLICT OF INTEREST

None declared.
